# Transcriptional responses to diets without mineral phosphorus supplementation in the jejunum of two high-yielding laying hen strains

**DOI:** 10.1016/j.psj.2024.104484

**Published:** 2024-11-02

**Authors:** Yosef A. Abitew, Henry Reyer, Frieder Hadlich, Michael Oster, Nares Trakooljul, Vera Sommerfeld, Markus Rodehutscord, Klaus Wimmers, Siriluck Ponsuksili

**Affiliations:** a Research Institute for Farm Animal Biology (FBN), Dummerstorf, Germany; b University of Hohenheim, Institute of Animal Science, Stuttgart, Germany; c University of Rostock, Faculty of Agricultural and Environmental Sciences, Rostock, Germany

**Keywords:** Dietary phosphorus, RNAseq, Jejunum mucosa, Laying hen, Maturation period

## Abstract

Phosphorus (**P**) is an essential mineral for all forms of life including laying hens, playing a crucial role in growth and efficient egg production. Recent studies suggest that current P recommendations might exceed the physiological demand, leading to unnecessarily high P excretions. This study on Lohmann Brown (**LB**) and Lohmann Selected Leghorn (**LSL**) laying hens (n=80; 10 replicates per strain, production period, and dietary group) investigates transcriptional changes in the jejunum, a critical intestinal segment for mineral absorption, in response to a diet either without (**P-**) or with (**P+**) a mineral supplement from monocalcium phosphate, administered over a 4-week period during the transition (15–19 weeks) or onset of laying (20–24 weeks). DESeq2 analysis of RNA sequencing data revealed that most differentially expressed genes (**DEGs**) varied between strains and age groups, with less pronounced effects from dietary mineral P content. The 19-week-old LB hens showed a stronger response to dietary mineral P removal, with transcripts affiliated with increased adaptation of the metabolism and decreased immune pathway activation. The identified pathways such as folate biosynthesis and p53 signaling, potentially link altered energy and amino acid metabolism (2-oxocarboxylic acid and arginine). Interestingly, genes involved in calcium transport (*CALB1*) and cellular signaling (*PRKCA, STEAP4*) along with tight junctions (*CLDN2*) were affected by complete removal of mineral P supplements, suggesting a promoted intestinal mineral uptake. Transcriptional regulation in the jejunum in response to low dietary mineral content is strain-specific when the laying phase begins, which may contribute to a physiological Ca:P ratio.

## Introduction

Phosphorus (**P**) is a fundamental mineral that serves as a building block for all forms of life and has a vital role in the growth and development of poultry. A high proportion of P in plant-origin feedstuff is not available for direct absorption as it is in the form of phytic acid, which can be hydrolyzed by poultry only to a limited extent due to a low endogenous phytase activity (Rodehutscord et al., 2022). Therefore, mineral P or exogenous phytases of microbial origin are routinely supplemented to poultry diets. Current dietary non-phytate P (**NPP**) recommendation for laying hens ranges from one to 4.5 g/kg depending on maturation period, and is assumed to be the same for LB and LSL despite their differences in mineral utilization (Li et al., 2017; Reyer et al., 2021). However, there is growing evidence that the current formulations of poultry feed contain mineral P levels that exceed requirements (Pongmanee et al., 2020; Rodehutscord et al., 2023), leading to unnecessarily high levels of mineral P being excreted in poultry manure. Excessive P excretions from poultry farming contribute to global P depletion and, in particular, pollute water bodies and ecosystems (Pope, 1991; Kyriazakis and Leinonen, 2016). Therefore, it is crucial for sustainable egg production and environmental sustainability to reduce the P content in the diet while maintaining production and hen welfare (Jing et al., 2018).

Two laying hen strains that are important in the global market, namely Lohmann Selected Leghorn (**LSL**) and Lohmann Brown-Classic (**LB**), have both demonstrated very high egg-laying performance throughout their production period (Habig et al., 2012). Despite having similar performances, these two strains distinctly differ in terms of immune traits, mineral utilization, body weight, as well as other phenotypical traits (Habig et al., 2012; Sommerfeld et al., 2020a; Schmucker et al., 2021). Our previous study on dietary calcium (**Ca**) and P supplementation in LB and LSL suggests that both strains may have differing Ca, P, and vitamin D requirements (Reyer et al., 2021). Sommerfeld and colleagues observed higher phytase activity and total phosphatase levels in LB compared to LSL (Sommerfeld et al., 2020a). In addition, LB and LSL hens have different intrinsic pathways to effectively cope with reduced P and Ca levels while ensuring mineral homeostasis and maintaining performance (Sommerfeld et al., 2020b; Iqbal et al., 2021; Omotoso et al., 2021).

In addition, not only the genetic background of the laying hen plays an important role in mineral utilization, but also the developmental stage. During the transition from the grower to the egg-laying phase, remarkable decrease in plasma P and a significant increase in Ca concentrations were observed, especially between 16 and 24 weeks of life (Sommerfeld et al., 2020a; Omotoso et al., 2023). Laying hens require a high Ca intake during the laying period for eggshell formation and this causes more Ca-phytate complex formation in the gut and endogenous phytate degradation is lower than in broilers (Rodehutscord et al., 2022). This prominent transformation was also confirmed at the molecular level of jejunal mucosa transcripts, such as ATPase Plasma Membrane Ca2+ Transporting 1 (*ATP2B1*), Calbindin 1 (*CALB1*), Sodium/Calcium Exchanger 1 (*NCX1*), Solute Carrier Family 20 Member 1 (*SLC20A1*), Solute Carrier Family 20 Member 2 (*SLC20A2*), and Solute Carrier Family 34 Member 2 (*SLC34A2*), with a dynamic pattern along the maturation from 16-24 weeks of age (Sommerfeld et al., 2020a; Ponsuksili et al., 2021). In fact, the jejunum mucosa plays a crucial role in the digestion and absorption of minerals and is a primary site of P absorption (Hurwitz and Bar, 1970). Absorption of P through the jejunum mucosa is a complex, tightly regulated mechanism involving both transcellular and paracellular pathways (Christakos et al., 2016). The transcellular absorption is mainly driven by parathyroid hormone (**PTH**), fibroblast growth factor 23 (**FGF-23**), and vitamin D3, and realized by specific proteins like NPt2 (*SLC34A2*) (Bacic et al., 2006). The paracellular transport pathway plays a supportive role in maintaining P homeostasis, especially when challenged with different diet conditions (Kiela and Ghishan, 2016; Knöpfel et al., 2019). Starting at the 16^th^ week, laying hens under commercial conditions are exposed to a significant increase in dietary Ca content, to which the animal, including its intestine, has to adapt.

We hypothesize that there is a complex interplay of diet, age, and genetic factors on gene expression patterns in the small intestine, i.e. the jejunum mucosa, which is a critical tissue for nutrient absorption and P utilization. In this study, P supply conditions were created by completely omitting the mineral P supplement from the diet (P-) compared to a diet with mineral P supplement from monocalcium phosphate (P+). The main objective of this study was to compare strains in a narrow maturation window from week 19 to week 24 in their P utilization through transcriptional changes in the gut induced by a low-P diet. The more challenging diet and shortened transition period from pullet to laying hen in both strains, compared to our previous study (Sommerfeld et al., 2020b) will facilitate a deeper understanding of biological networks and gene expression as influenced by diet, maturation, and genetic background.

## Materials and methods

This study was part of the interdisciplinary Research Unit P-Fowl: Inositol phosphates and *myo*-inositol in the domestic fowl: Exploring the interface of genetics, physiology, microbiome, and nutrition (https://p-fowl.uni-hohenheim.de/).

### Experimental design

The study comprised a 2 × 2 × 2-factorial arrangement, including hen strain (LB – Lohmann Brown; LSL – Lohmann Selected Leghorn), production periods (19 weeks; 24 weeks), and diet (with and without mineral P supplements in form of monocalcium phosphate). A total of 40 LB and 40 LSL hatchlings were obtained from Lohmann Tierzucht GmbH (Cuxhaven, Germany) and raised in floor pens with deep litter bedding, following the standardized procedures of the station. During two time periods (weeks 15 and 20), hens were housed in metabolic units for 4 weeks and fed their respective experimental diets. In the first group that was slaughtered at 19 weeks, hens were placed in metabolic units at 15 weeks and fed a sequence of three diets with or without mineral P supplementation (Supplementary Table S1). The feeding regimen for hens leading up to slaughter at 19 weeks was structured across three diets, each offered with two phosphorus levels, low (P-) or high (P+). From weeks 15 to 16, hens received the developer diet with either P- or P+. During weeks 16 to 17, they transitioned to the prelayer diet, maintaining the designated P level. Finally, from weeks 17 to 19, hens were fed the layer diet, continuing with P- or P+ as initially assigned.

In the second group, the hens were similarly raised on the developer, prelayer and layer diets until 20 weeks of age and received a mineral P supplementation. From week 20, the start of the experimental diversion and the placement into metabolic units, the hens continued to receive the laying hen diet until week 24, but now with either P- or P+ phosphorus supplement (Supplementary Table S1). At the end of each period, corresponding to early maturation (week 19) and the onset of egg laying (week 24), the hens were killed for sampling (Sommerfeld et al., 2024).

During the study period, the hens received maize-soybean meal-based diets without phytase supplement, and the diets were formulated to contain all nutrients at the recommended levels, except P (Table S1). The diets contained no exogenous enzymes, functional additives, or pre-/probiotics, but were supplemented with DL-Methionine and L-Lysine sulfate. The diet group without mineral phosphorus supplementation (P-) was compared to the group with mineral phosphorus from monocalcium phosphate (P+), resulting in a 1 g/kg difference in NPP between the groups (Table S1). The low-P diet (P-) contained no mineral phosphorus supplements and had a total phosphorus content of 3.1 g/kg dry matter (DM), corresponding to 1.3 g/kg NPP, in the layer feed during weeks 17–24, including at the sampling points at the end of weeks 19 and 24. In contrast, the P+ diet had a phosphorus content of 4.1 g/kg DM, corresponding to 2.3 g/kg NPP (Table S1).

Ten hens per strain and production period and dietary group were sampled (n=80 hens). The hens were stunned by 35% CO_2_, 35% N_2_, and 30% O_2_ and then killed by exsanguination. In each period, sampling took place on 2 consecutive days between 09:00 h and 14:00 h. Jejunum samples of approximately 2-3 cm were collected from approximately 3 cm distal to the duodenal loop. The samples were cut open and the mucosa was thoroughly rinsed with a 0.9 % NaCl solution. The scrapings of small intestinal mucosa (jejunum) samples were immediately frozen in liquid nitrogen and stored at -80°C until RNA extraction.

### RNA extraction, library preparation, and sequencing

Approximately 50 mg of jejunal mucosa tissue from 80 samples was used for RNA isolation using TRI reagent according to the manufacturer's protocol (Sigma-Aldrich Chemie GmbH, Taufkirchen, Germany). DNase treatment was performed to remove DNA fragments. RNA quality and quantity were determined using NanoDrop ND-2000 (Peqlab, Erlangen, Germany), a Qubit Fluorometer (Thermo Fisher Scientific, Dreieich, Germany) and a Bioanalyzer 2100 (Agilent Technologies, Waldbronn, Germany). RNA quality was assessed using the RNA integrity number (**RIN**), with an average RIN of 7.7 ± 0.6 (mean ± SD) (Table S2). The total RNA was used for mRNA sequencing library preparation using the Illumina Stranded mRNA library preparation kit according to the manufacturer's protocol (Illumina, San Diego, CA, USA). Briefly, poly-A-tailed mRNA molecules were enriched using poly-T oligo attached magnetic beads followed by fragmentation and first-strand cDNA synthesis with random primers. Each DNA library was barcoded with a different indexing adapter to enable a parallel sequencing by pooling multiple samples. The DNA libraries were quality checked using a DNA 1000 chip (Agilent Technologies) and quantified for molar concentration using the Qubit dsDNA HS assay kit (Invitrogen, Darmstadt, Germany). Sequencing was performed on the Nextseq 2000 (Illumina, San Diego, CA, USA) at the sequencing facility of the Research Institute for Farm Animal Biology (**FBN**), Dummerstorf, Germany.

### Pre-processing sequencing reads

Raw sequencing reads were collected from 80 jejunum mucosa samples and converted to FASTQ files from the base call (**BCL**) file format using bcl2fastq software (version 2.2.0). Adapter-like sequences were removed using Trim Galore (version 0.6.7). The reads were then subjected to FASTQC (version 0.11.9) for quality control, and the raw reads were pre-processed by removing low-quality reads with a mean Q-score of less than 20 and short length reads of less than 30 base pairs. The reads that passed quality control were considered for downstream analysis.

### Alignment, mapping, and removing batch effects

HISAT2 version 2.2.0, an alignment program for mapping high-throughput next-generation sequencing reads, was used in the nf-core/rna-seq pipeline with default parameters (Kim et al., 2019). On average, 45.35 million reads per sample were mapped to the chicken reference genome GRCg6a. Read counts were assigned for features using htseq-count (Anders et al., 2015), which was developed in the framework of the HTSeq Python tool with the default “union” mode (version 2.0.2) as defined by the Ensembl GTF file. To ensure that no additional undesired effects were present after adjusting for strain, diet, and age in our model, we employed the SVA package (version 3.46.0) and its svaseq function. This allowed estimating batch variables (**BV**s) that accounted for potential batch effects. After normalizing the count matrix, *svaseq* was applied to estimate the factors that accounted for the batch effects. The first two BVs (BV1 and BV2) were included in the final model. With the two batch variables calculated (BV1 and BV2), the final model for differential expression analysis included strain, diet, age, and batch variables (BV1 and BV2).

### cDNA synthesis and quantitative real-time PCR

For cDNA synthesis, 200 ng of mRNA was mixed with 1 μL of Reverse Transcription Master Mix (Fluidigm PN 100-6297) in a total volume of 5 μL. The reaction was incubated at 25 °C for 5 minutes, 42 °C for 30 minutes, followed by 85 °C for 5 minutes. The resulting cDNA was used for quantitative polymerase chain reaction (**qPCR**). cDNA samples were then subjected to qPCR using the Fluidigm BioMark HD System (Fluidigm Corporation, CA, USA). Specific target amplification (**STA**) was performed according to the manufacturer's recommendations using PreAmp Master Mix (Fluidigm PN 1005581). Fluidigm quantitative measurement runs were carried out with 96.96 dynamic arrays (Fluidigm Corporation, CA, USA). The array chips were placed in the BioMark Instrument for polymerase chain reaction (**PCR**) at 95 °C for 10 minutes, followed by 30 cycles at 95 °C for 15 seconds and 60 °C for 1 minute. Data were analyzed using real-time PCR analysis software on the BioMark HD instrument (Fluidigm Corporation, San Francisco, CA). The internal controls included four reference genes: *GAPDH, ACTB, RPL13*, and *TBP* were used (Zhang et al., 2018). The expression levels of these reference genes were checked to ensure that they did not differ between groups. The geometric mean of the Ct values for the four reference genes (*GAPDH, ACTB, RPL13,* and *TBP*) was used to calculate the ΔCt for each target gene in each sample. The normalized data using the 2^−ΔCt^ method was further used for qPCR data analysis. Data were analyzed using SAS 9.4 statistical software (SAS Institute) and the mixed procedure. The statistical model included effects of strain, age and diet as well as strain*age*diet interaction. Father/Rooster was used as a random effect. Post hoc Tukey–Kramer method was used for multiple comparison adjustments. Results were reported as least-squares means (**LSmeans**) with standard error (**SE**) and considered to be statistically significant if p < 0.05.

### Data analysis

Downstream gene-level RNA-seq analysis was carried out using the *DESeq2* package (Love et al., 2014a) in the R environment (R Foundation for Statistical Computing, Vienna, Austria; URL https://www.R-project.org). The *arrayQualityMetrics* (**AQM**) package version 3.56.0 from the Bioconductor project was used to detect outliers based on sample-to-sample distances, distributions of counts, and differences between the log2 counts of a gene in one sample and the corresponding log2 average across all samples (Kauffmann et al., 2009). The thresholds for defining outliers were set to the default values in the AQM package (version 3.60.0). No outliers were detected in any of the metrics. Differential expression analysis was performed on data from 13,332 genes. Principal component analysis (**PCA**) was carried out using *DESeq2* following variance stabilizing transformation (**VST**) of data. To enhance the statistical power for identifying differentially expressed genes, we performed pre-filtering to retain only data from genes with ten or more counts in at least five samples (Love et al., 2014a).

### The following statistical full model was used

$$yijk\;=\;\mu \;+\;\alpha i\;+\;\beta j\;+\;\gamma k\;+\;BV1\;+\;BV2\;+\;\varepsilon ij_{k}$$

Where, y_ijk_ = response vector; µ = the overall mean; α_i_ = strain effect (fixed); β_j_ = effect of dietary P (fixed); γ_k =_ age effect (fixed); *BV1* = batch variable 1; *BV2* = batch variable 2; and ε_ijk_ = vector of residual errors. DESeq2 uses a Wald test with *p-values* adjusted for multiple testing using the Benjamini and Hochberg procedure (Love et al., 2014b). Genes were considered as significantly differentially expressed meeting the criteria of *p-value* < 0.01 for diet factor and Benjamini-Hochberg adjusted *p-value* < 0.01 for strain and age factors. Two distinct approaches were used to conduct differential expression analysis (DEGs). Firstly, a full model, as described above, was applied across all LSL and LB hens within each dietary and age group to identify significant main effects. Secondly, a statistical model was designed to reveal DEGs in the contrasts of diets within each strain at the same time point, such as LBweek19P+ vs. LBweek19P-, LSLweek19P+ vs. LSLweek19P-, LBweek24P+ vs. LBweek24P-, and LSLweek24P+ vs. LSLweek24P-. This model included BV1 and BV2 as fixed effects. This approach is particularly useful for assessing gene expression changes within each strain under various comparisons and for minimizing sample variability.

Ensembl gene identifiers from expressed genes were converted to corresponding official gene symbols using bioDBnet: the biological database network (Mudunuri et al., 2009) with the default options. The VolcaNoseR tool, accessed via the website from https://goedhart.shinyapps.io/VolcaNoseR/ were utilized to visualize the transcriptional expression profiles and identify the top significant genes. Gene ontology (**GO**) analysis and Kyoto Encyclopaedia of Genes and Genomes *(****KEGG***) pathway enrichment analysis were analyzed using DAVID and functional annotation bioinformatics tools. To identify enriched biological pathways among DEGs a Benjamini-Hochberg adjusted *p-value* corresponding to a false discovery rate (**FDR**) < 0.01 for strain and age comparisons and a nominal *p-value* < 0.05 for diet groups were used (Huang et al., 2009a; b).

## Results

**Fig. 1:**
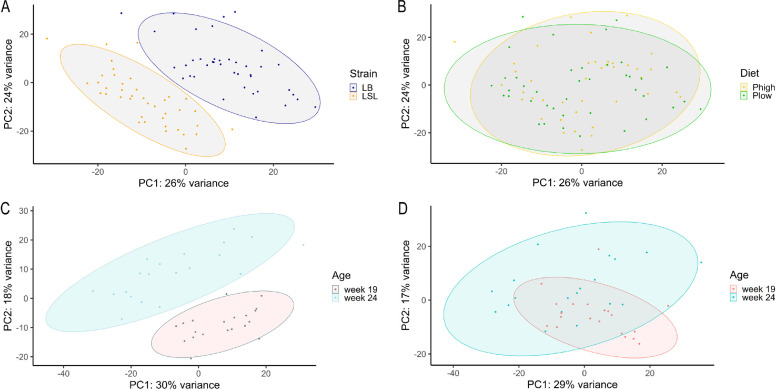
Principal component (PC) analysis of the jejunal RNA sequencing data obtained from LB and LSL laying hens fed a diet lacking any mineral P supplement (P-) or a diet with mineral P supplement (P+) sampled at early maturation (19 weeks) and onset of lay (24 weeks). The main experimental effects of laying hen strain (A) and dietary P supply (B) as well as developmental stages within LSL (C) and LB (D) strains are displayed. The ellipses represent the 95% confidence interval for the respective groups.

The analysis of the 80 hen samples using RNA sequencing resulted in an average of 45.35 million reads per sample, which could be assigned to the chicken reference genome, with 75% of reads aligned and 100% mapped (Table S2). The resulting 13,332 transcripts that passed quality control were used for further analysis. The principal component analysis revealed a clear separation between strains (Fig. 1A) but not due to mineral P level (Fig. 1B). For the age groups, there was a clear distinction in gene expression patterns within the LSL strain between week 19 and week 24 (Fig. 1C). Age groups in LB, on the other hand, had a large overlap for the first two principal components and showed no clear separation (Fig. 1D).

### Differentially expressed genes of the main effects strain, production period, and diet

**Fig. 2:**
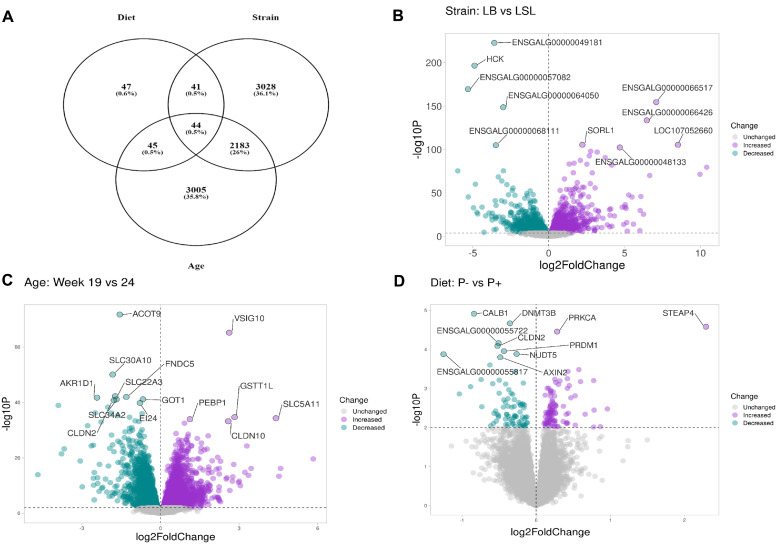
Differentially expressed genes (DEGs) derived from jejunum of LB and LSL laying hens fed a diet lacking any mineral P supplement (P-) or a diet with mineral P supplement (P+) sampled at early maturation (19 weeks) and onset of lay (24 weeks). Venn diagram of DEGs representing the effects of laying hen strain, age, and dietary P supply (A). Volcano plot visualization of the identified DEGs associated with laying hen strain, adj-p < 0.01 (B), hen age, adj-p < 0.01 (C), and dietary P supply, p < 0.01 (D); Increased expression is displayed in purple: LB > LSL, wk19 > wk24; P+ > P-; Decreased expression is displayed in green: LB < LSL, wk19 < wk24; P+ < P-.

Differential gene expression analysis was conducted between LB and LSL hens to compare the effect of P diet groups at two different production periods. In total, 8,393 transcripts were statistically significant at least in one of the main effects of the factors strain, age, and diet (Fig. 2A). The differential expression results for the main effects are presented in the supplementary Table S2. Strain exhibited the most significant changes, followed by age and diet. Specifically, 44 transcripts showed significant differential expression across all factors, while 3,028, 3,005, and 47 transcripts were exclusively differentially expressed between strains, age, and diet, respectively.

In the jejunum mucosa, 5,296 genes exhibited significant differential expression between strains, with 2,436 genes showing increased abundance and 2,860 genes showing decreased abundance in the LB strain compared to LSL. Examples of genes with higher abundance of transcripts in LSL include Carbohydrate Sulfotransferase 14 (*CHST14*), Hemopoietic Cell Kinase Proto-Oncogene, Src Family Tyrosine Kinase (*HCK*), Tetratricopeptide Repeat Domain 9 (*TTC9*), and novel transcripts (*ENSGALG00000057082* and *ENSGALG00000064050*), while genes such as Sortilin Related Receptor 1 (*SORL1*), LOC107052660, Proprotein Convertase Subtilisin/Kexin Type 4 (*PCSK4*), and novel transcripts *(ENSGALG00000066517* and *ENSGALG00000066426*) were more abundant in the LB strain (Fig. 2B). Around 5,277 genes were significantly differentially expressed between week 19 and week 24 across both strains, with 2,769 increased and 2,508 decreased at week 19 compared to week 24. Exemplary genes like Solute Carrier Family 30 Member 10 (*SLC30A10*), Claudin 2 (*CLDN2*), Solute Carrier Family 22 Member 3 (*SLC22A3*), Glutamic-Oxaloacetic Transaminase 1 (*GOT1*), and Aldo-Keto Reductase Family 1 Member D1 (*AKR1D1*) were significantly increased at week 24 while Solute Carrier Family 5 Member 11 (*SLC5A11*), Claudin 10 (*CLDN10*), Phosphatidylethanolamine Binding Protein 1 (*PEBP1*), V-Set Immunoregulatory Receptor 10 (*VSIG10*), and Glutathione S-Transferase Theta 1 Like (*GSTT1L*) showed lower abundance (Fig. 2C). Moreover, 177 differentially expressed transcripts were observed between dietary groups at *p-value* < 0.01 (Fig. 2A). Of these, 98 genes showed an increased expression (P- > P+) in the P+ diet group, whereas 79 genes were decreased (P- < P+). Interesting transcript changes based on the P- diet were observed, including *CALB1*, DNA Methyltransferase 3 Beta (*DNMT3B*), *CLDN2*, PR/SET Domain 1 (*PRDM1*), and Nudix Hydrolase 5 (*NUDT5*) showing an increased abundance, while STEAP Family Member 4 (*STEAP4*), Protein Kinase C Alpha (*PRKCA*), Coproporphyrinogen Oxidase (*CPO*), DNA Polymerase Gamma 2 Accessory Subunit (*POLG2*), and Solute Carrier Family 5 Member 11 (*SLC5A11*) decreased in response to the removal of mineral P in the diet (Fig. 2D; Table S3).

**Fig. 3:**
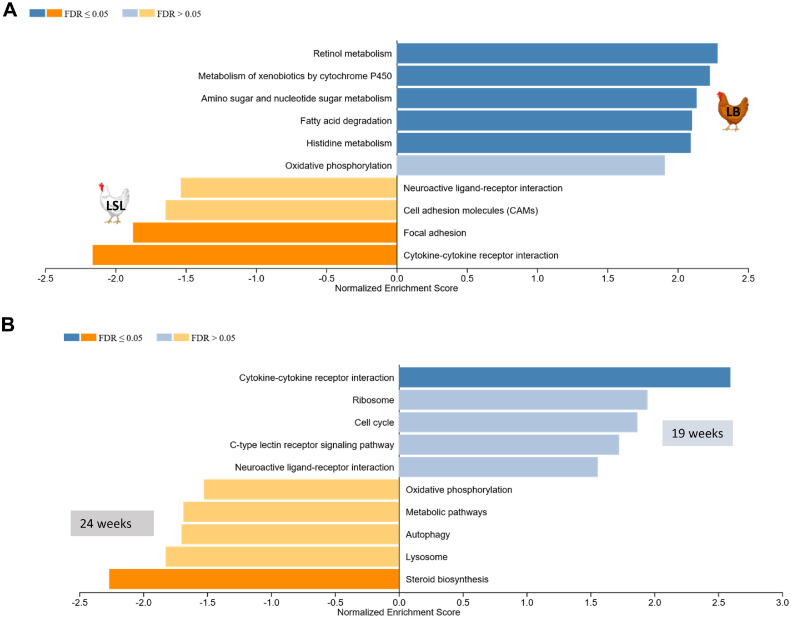
Enriched *KEGG* pathways derived from the differentially expressed genes (DEGs) with higher expressions observed in LB (blue) or LSL (yellow) when comparing LB and LSL laying hen strains (A), as well as enriched *KEGG* pathways based on the identified DEGs with higher expressions observed at 19 weeks (blue) or 24 weeks (yellow) when comparing maturation effects, i.e., the two age groups (19 vs. 24) (B).

Based on identified DEGs, gene ontology of biological processes (**BP**) and *KEGG* pathway analyses were performed (Table S4). Among the DEGs between strains, lipid transport, epithelial cell differentiation, inflammatory response, and phosphatidylinositol-3-phosphate biosynthetic processes were enriched in biological processes. The *KEGG* pathways identified included cell cycle, metabolic pathways, DNA replication, histidine metabolism, and inositol phosphate metabolism. Enrichment of DEGs in LB was observed in metabolism of xenobiotic by cytochrome P450, retinol metabolism, metabolism of amino and nucleotide sugars, histidine metabolism, and fatty acid degradation, while LSL was more pronounced in cytokine-cytokine receptor interaction and focal adhesion (Fig. 3A). For age effects, biological processes such as tricarboxylic acid cycle, carbohydrate metabolic process, inflammatory response, and protein phosphorylation were also enriched processes along with nucleotide metabolism, galactose metabolism, carbon metabolism, and steroid biosynthesis pathways (Table S4). Cytokine-cytokine receptor interaction was enriched at 19 weeks, whereas steroid biosynthesis was enriched at 24 weeks (Fig. 3B). The *KEGG* pathway analysis of DEGs in the diet group from the main effects revealed their involvement in various biological processes (Table S4). These processes include folate biosynthesis, p53 signaling pathway, 2-oxocarboxylic acid metabolism, and arginine biosynthesis.

**Fig. 4:**
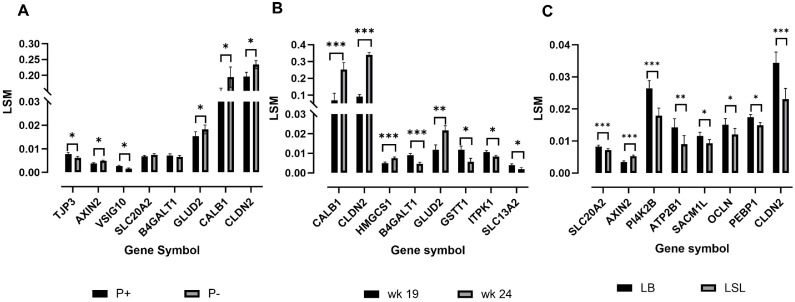
Gene expression levels validated by qPCR. Analysis are based on least square means (LSM). Comparisons are displayed for effects by strain (A), age (B), and diet (C) (*** padj ≤ 0.005, ** padj ≤ 0.01, * padj ≤ 0.05). Transcripts were selected based on their functional relevance to P and Ca transport mechanisms, or their involvement in mineral utilization pathways.

Real-time qPCR was employed to validate the expression patterns of 24 genes (Table S5), chosen based on their biological function in mineral transport and absorption. Gene expression levels validated by qPCR showed differential expression due to the effect of strain (Fig. 4A), age (Fig. 4B), and diet (Fig. 4C). Spearman correlations between RNA-seq and qPCR results ranged from 0.38 to 0.89, all showing statistical significance for the selected candidate genes (p < 0.05). Thus, the qPCR analyses confirm the reliability of the RNA-seq statistical workflow.

### Diet comparison within each strain and age

**Fig. 5:**
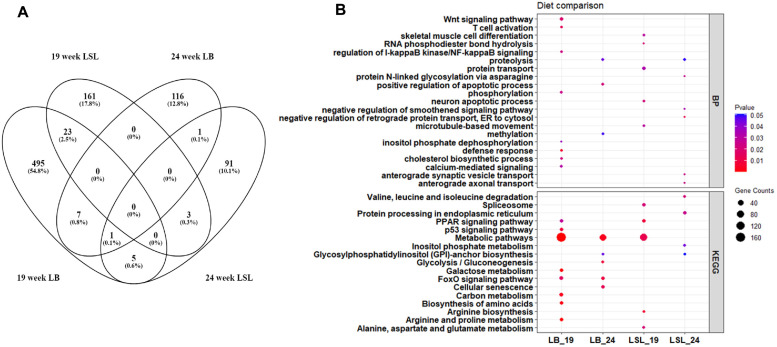
Venn diagram of the number of differentially expressed genes (DEGs) identified by diet-specific comparisons within LB and LSL laying hens at 19 and 24 weeks of age fed a diet lacking any mineral P supplement (P-) or a diet with mineral P supplement (P+) (p < 0.05) (A). *KEGG* pathways and biological processes enriched by DEGs derived from comparisons of diet (P+ and P-) at specific ages (19 and 24 weeks) for each laying hen strain (LB and LSL) (p < 0.05) (B).

We further considered DEGs in the contrasts of diets within each strain at the same time point, including the following comparisons: LBweek19P+ vs LBweek19P-, LSLweek19P+ vs LSLweek19P-, LBweek24P+ vs LBweek24P-, and LSLweek24P+ vs LSLweek24P-. In total, 903 transcripts were found to be significant in at least one of these combinations at a *p-value* threshold of less than 0.01 (Fig. 5A; Table S6).

The specific comparison of the hens receiving divergent dietary P levels within the individual strains (n = 40) showed that 531 genes were found to be differentially expressed in the LB strain at week 19, while 125 genes were differentially expressed at 24 weeks of age (Fig. 5A). In LSL hens, analysis at week 19 showed 187 DEGs, while 101 DEGs were revealed at week 24. The overlay of the strain-specific DEGs revealed no genes that were commonly identified in LB and LSL birds at both time points in response to the variable dietary P supply (Fig. 5A). The highest significant DEGs between diet at week 19, including *CALB1*, Sclerostin Domain Containing 1 (*SOSTDC1*), Leucine Rich Transmembrane Neuronal 2 (*LRTM2*), *CLDN2*, and *AKR1D1,* were found to have higher expression levels in the both LB and LSL hens fed the P- diet compared to the control diet (Table 1). These genes are involved in various cellular processes, such as Ca transport and uptake (*CALB1*), control cell proliferation (*SOSTDC1*), lipid transport (*LRTM2*), passive transport (*CLDN2*), and bile acid metabolism (*AKR1D1*). Interestingly, some genes, including *PRKCA* and RB Transcriptional Corepressor 1 (*RB1*) showed lower expression levels in the both LB and LSL hens on the P- diet compared to P+. While Glutamic-Pyruvic Transaminase 2 (*GPT2*), Ro60 Associated Y RNA (*RNT*), and LOC417131 showed significant lower expression on the P- diet only in LSL strain. Genes such as Ectonucleoside Triphosphate Diphosphohydrolase 8 Like 2 (*ENTPD8L2*), and Lysosomal Protein Transmembrane 4 Beta (*LAPTM4B*) showed increased expression levels, while ST3 Beta-Galactoside Alpha-2,3-Sialyltransferase 4 (*ST3GAL4*) and Solute Carrier Family 2 Member 8 (*SLC2A8*) decreased in LB hens fed P- diet at week 24 (Table 1). Conversely, the expression levels of RUN And TBC1 Domain Containing NL (*RUBCNL*) and Complement C8 Alpha Chain (*C8A*) were lower in P- diet fed LSL hens, while Yolk Lipoprotein Complex 8 (*YLEC8*) and Tubulin Alpha 1a (*TUBA1A*) showed higher transcript abundance compared to P+ at week 24 (Table 1).

**Table 1:** Top annotated DEGs indicating the effect of low dietary P content within each laying hen strain (LB and LSL) in the jejunum at 19 and 24 weeks of age.

		**LB**			**LSL**			
**Gene**^1^	**baseMean**^2^	**Log2FC**^3^	***p-value***	**padj**^4^	**Log2FC**^3^	***p-value***		**Padj**^4^
***Week 19***								
CALB1	16733.5	-1.83	2.73E-07	0.0033	-1.24	0.0005	0.3505	
PRKCA	572.2	0.57	7.08E-06	0.0297	0.35	0.0063	0.6813	
RB1	334.8	0.63	7.65E-06	0.0297	0.36	0.0115	0.7440	
SOSTDC1	29.1	-0.99	1.18E-05	0.0297	-0.39	0.0781	0.8870	
LRTM2	42.3	-1.49	1.41E-05	0.0297	-0.86	0.0198	0.7731	
CLDN2	10773.5	-0.85	0.0005	0.0877	-1.11	7.17E-06	0.0319	
AKR1D1	1876.8	-0.64	0.0533	0.4077	-1.45	1.58E-05	0.0429	
GPT2	1486.2	0.04	0.8228	0.9575	0.64	7.30E-05	0.1622	
RNF170	32.9	0.34	0.0621	0.4251	0.75	8.86E-05	0.1639	
LOC417131	108.7	0.29	0.0638	0.4288	0.64	9.83E-05	0.1639	
***Week 24***								
ENTPD8L2	320.5	-1.33	1.2E-06	0.0160	0.18	0.4873	0.9693	
ST3GAL4	4739.5	4.46	4.2E-05	0.2537	-0.52	0.6216	0.9754	
SLC2A8	152.1	0.48	5.7E-05	0.2537	-0.08	0.4746	0.9693	
LAPTM4B	151.7	-1.57	1.1E-04	0.2915	-0.18	0.6482	0.9776	
YLEC8	91.5	0.73	0.5542	0.9731	20.98	0.0000	0.0000	
RUBCNL	108.6	-0.21	0.3678	0.9702	-0.92	0.0001	0.1642	
C8A	28.1	0.81	0.3418	0.9702	-3.58	0.0001	0.2401	
TUBA1A	4243.4	-0.16	0.2867	0.9702	0.57	0.0001	0.2401	

1 *CALB1*, Calbindin 1; *PRKCA*, Protein Kinase C Alpha; *RB1*, RB Transcriptional Corepressor 1; *SOSTDC1*, Sclerostin Domain Containing 1; *LRTM2*, Leucine Rich Transmembrane Neuronal 2; *CLDN2*, Claudin 2; *AKR1D1*, Aldo-Keto Reductase Family 1 Member D1; *GPT2*, Glutamic-Pyruvic Transaminase 2; *RNF170*, Ring Finger Protein 170; *LOC417131*, Uncharacterized LOC417131; *ENTPD8L2*, Ectonucleoside Triphosphate Diphosphohydrolase 8 Like 2; *ST3GAL4*, ST3 Beta-Galactoside Alpha-2,3-Sialyltransferase 4; *SLC2A8*, Solute Carrier Family 2 Member 8; *LAPTM4B*, Lysosomal Protein Transmembrane 4 Beta; *YLEC8*, Yolk Lipoprotein Complex 8; *RUBCNL*, RUN And TBC1 Domain Containing NL; *C8A*, Complement C8 Alpha Chain; *TUBA1A*, Tubulin Alpha 1a.

2 The average of normalized counts across all samples from the DESeq2 analysis;

3 Logarithmic representation of the fold change with positive values indicating P+ > P- and negative values indicating P+ < P-;

4 False discovery rate (FDR)-adjusted *p-value* using the Benjamini-Hochberg procedure.

**Fig. 6:**
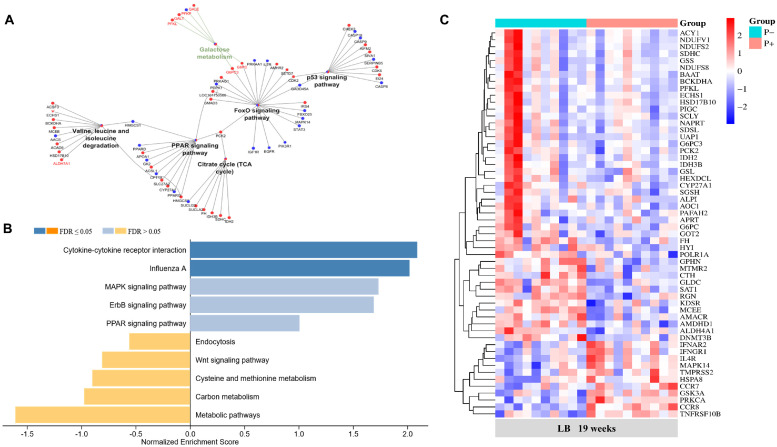
Significantly enriched *KEGG* pathways in the diet comparison in LB hens at 19 weeks, their respective genes and their interaction, blue nodes indicating increased expression and enrichment due to the P+ diet and red nodes indicating increased expression and enrichment due to the P- diet (p < 0.05) (A). *KEGG* pathways of enriched transcripts upregulated due to the P- (yellow) and P+ (blue) diet of LB hens at 19 weeks (B). Heatmap of significantly expressed genes involved in the identified enriched pathways, i.e., cytokine-cytokine receptor interaction, influenza A, metabolic pathways, and carbon metabolism in 19-week old LB hens comparing P- vs. P+ diet groups (C).

The results from *KEGG* and GO enrichment analyses were depicted using bubble maps, aiding in the comprehension of enrichment pathways and functions of diet-induced DEGs across different age and strain combinations (Fig. 5B; Table S7). In LB, GO analysis highlighted significant involvement in various BPs, notably Wnt signaling pathways, T cell activation, cholesterol biosynthesis, and defense response at 19 weeks. *KEGG* analysis emphasized metabolic pathways as the primary enrichment, particularly prominent at 19 weeks in LB hens (Fig. 5B). In addition, diet-induced DEGs were also enriched in galactose metabolism, amino acid biosynthesis, citrate cycle (TCA cycle), p53 signaling pathway, FoxO signaling pathway, and PPAR signaling pathway in LB hens at 19 weeks (Fig. 6A). In LSL hens at 19 weeks, the dietary effects predominantly enriched processes such as protein transport, arginine biosynthesis, spliceosome activity, and PPAR signaling pathway. However, at 24 weeks of age, LB hens exhibited fewer transcript changes attributable to dietary effects compared to 19 weeks, though metabolic pathways remained significantly altered. This contrasted with LSL hens at the same age, where metabolic pathway alterations were less pronounced (Fig. 5B). At 19 weeks of LB hens, we further delved deeper into dietary alterations by conducting an enrichment analysis of upregulated and downregulated pathways between the P+ and P- diet groups. We found that pathways associated with cytokine-cytokine receptor interaction, Influenza A, and MARK signaling were more enriched in the P+ group. Conversely, the P- group exhibited enrichment in metabolic pathways, carbon metabolism, and cysteine and methionine metabolism (Fig. 6B). About 45 transcripts belonging to metabolic pathways were upregulated in the P- group, while 11 transcripts enriched in cytokine-cytokine receptor interaction and influenza A were upregulated in the P+ group in LB at 19 weeks (Fig. 6C).

## Discussion

P is essential for life but is a limited resource. Excess P in poultry feed leads to high P excretion, contributing to environmental issues. Growing evidence indicates that current poultry feed formulations contain mineral P levels exceeding requirements (Pongmanee et al., 2020; Rodehutscord et al., 2023). Our previous study showed only subtle changes in physiological, metabolic, and molecular parameters when comparing a diet with the recommended phosphorus (P) level to a diet with 20% lower P content (Sommerfeld et al., 2020; Reyer et al., 2021). This led us to hypothesize that a wider difference between the dietary phosphorus levels might be necessary to detect a diet-related effect. Therefore, in the current study, we used diets with even lower phosphorus levels and no mineral phosphorus supplementation at all (2.3 g/kg NPP VS 1.3 g/kg NPP). From the same study we found that body weight (BW) differed significantly between hen strains and production periods, but no effect of dietary P level on BW was observed (Sommerfeld et al., 2024). P intake differed significantly between strains (LB > LSL) and diets (P+ > P-), though P excretion did not differ between the dietary groups. Finally, P retention was significantly higher in LB hens and in the P+ diet group, suggesting more efficient utilization of the mineral P supplement (Sommerfeld et al., 2024).

The principal component analysis showed a clear separation between strains and age groups within LSL. However, there was no clear separation based on the first two components when considering the diet groups, suggesting less pronounced effects from removing mineral P from the diet. The strain difference between the LB and LSL hens could be attributed to genetic factors formed by selective breeding for high egg production over many years in both layer strains (Habig et al., 2012). LB hens, genetically closer to broiler breeds, may have higher feed drive and metabolic requirements than LSL hens, as confirmed in this study (Malomane et al., 2019). For instance, a study by Rubin et al., (2010) demonstrated selective sweeps of favorable alleles and candidate mutations that have played a role in the domestication of chickens and their specialization into layer strains. Our results show a greater effect of strains than aging and diet, confirming our previous studies, which were based on both physiological and molecular analyses (Omotoso et al., 2021; Iqbal et al., 2022; Ponsuksili et al., 2023). Enrichment in metabolic and energy metabolism pathways was observed in the jejunum of LB, while immune and focal adhesion was prominent in LSL, which confirms our previous study (Reyer et al., 2021). Focal adhesions are large protein complexes consisting of vinculin, talin, paxillin, zyxin, and α-actinin (Rikitake and Takai, 2011). They connect cells to the extracellular matrix (**ECM**) via integrins, supporting cell anchorage and migration, particularly important for intestinal enterocytes and immune cells (Uni et al., 2000). This function of the ECM is likely crucial for the observed differences in body weight, feed intake, and mineral utilization between LB and LSL hens, as previously shown by our study (Ponsuksili et al., 2023). These differences were accompanied by changes in molecular traits, suggesting an influence on metabolic pathways in LB hens and the immune system in LSL hens.

At week 19, genes involved in immune signaling pathways, gut cellular processes, and immune cells, such as Interleukin 17A (*IL17A*), Interleukin 17F (*IL17F*), TNF Receptor Superfamily Member 13C (*TNFRSF13C*), C-X-C Motif Chemokine Receptor 5 (*CXCR5*), and Interleukin 34 (*IL34*) showed an increased mRNA abundance. At week 24, the focus seems to shift. Genes related to steroid biosynthesis and energy metabolism, like Methylsterol Monooxygenase 1 (*MSMO1*), Sterol-C5-Desaturase (*SC5D*), Hydroxysteroid 17-Beta Dehydrogenase 7 (*HSD17B7*), NAD(P) Dependent Sterol Dehydrogenase Like (*NSDHL*), and Sterol O-Acyltransferase 1 (*SOAT1*), became more expressed, indicating a shift in physiological priorities from immune parameters to reproductive activities as hens’ transition from the maturation phase to the laying period (Johnston et al., 2012). At 19 weeks, the intestinal epithelium adapts to the changed nutrient supply in preparation for egg production, whereby intestinal integrity and immune function are a priority. By 24 weeks, as laying began, there is a shift in metabolism towards processes vital for egg formation and energy production which can also be detected by the corresponding regulations in the intestine (Shi et al., 2024). Another study demonstrated that there were disturbances of lipid metabolism, energy production and oxidation resistance of the intestine of laying hens in the late phase of production as compared to those at peak production (Wang et al., 2019). Our findings, along with those of (Schmucker et al., 2021), suggest that egg-laying may lead to a reduction in certain immune cells, indicating a modulation of the immune system to prioritize resources during this crucial production phase (Schmucker et al., 2021).

The differences in metabolic pathways were also evident when comparing dietary groups across the data set, with significant differences found in folate biosynthesis, p53 signaling, 2-oxocarboxylic acid metabolism and arginine biosynthesis. Folate acts as a donor of one-carbon units essential for methylation reactions and is involved in DNA synthesis, cell division, and amino acid metabolism (Fournier et al., 2002). Previous studies indicated a number of mechanisms by which epigenetic changes regulate gut development and are controlled by the microbiome (Yu et al., 2015). The identification of further significant DEGs in the jejunum mucosa in the diet group comparison (P- vs. P+), such as *CALB1, PRKCA, STEAP4, CLDN2, PRDM1*, and *NUDT5*, provides valuable insights into the molecular mechanisms underlying mineral absorption in the small intestine. The *CALB1* gene encodes calbindin-D28K, a Ca-binding protein that plays a crucial role in the transcellular Ca absorption pathway in the small intestine, predominantly localized at the brush border membrane in the duodenum, jejunum, and ileum of laying hens (Bar, 2009). Maintaining homeostasis of Ca2+ influx is the main function of *CALB1* (Gloux et al., 2019). In our study, *CALB1* showed an increased expression in the P- diet compared to the control diet and was particularly different at week 19 in both strains. This coincided with a trend towards lower luminal calcium concentrations in the jejunum of P- compared to P+ animals (Sommerfeld et al., 2024). At 24 weeks, there was no difference in *CALB1* transcript level between the diet groups. This could represent the adaptive response to the high Ca content and the shift in the Ca:P ratio in the diet with regard to the maintenance of Ca homeostasis and an increased Ca requirement for eggshell formation during the onset of laying (Shet et al., 2018). Previous studies also reported an increased expression of *CALB1* in laying hens and broilers when fed a low P diet (Bar et al., 1990; Nie et al., 2013; Rousseau et al., 2016). Most common genes encoding for P transport and utilization were found to be affected by the diets. For instance, *SLC20A1, SLC20A2, SLC34A2, TJP2*, and *TJP3* had an increased expression for P- diet compared to the P+ group, indicating an active process to maintain P levels for physiologic functions. The upregulation of phosphorus transport genes in the phosphorus-deficient (P-) group suggests that the body is compensating for reduced phosphorus intake, possibly indicating a state of subclinical phosphorus deficiency. Despite variations in P intake between strains and diets, P excretion remained consistent, suggesting efficient phosphorus retention in the P- group (Sommerfeld et al., 2024). The P+ diet and LB hens demonstrated higher phosphorus retention, indicating more efficient phosphorus utilization under these conditions (Sommerfeld et al., 2024). This supports the idea that the body enhances phosphorus absorption when dietary supply is limited.

The genes encoding *SLC34A2, SLC20A2, SLC20A1*, and *SLC34A3* mediate sodium-coupled transport of P (Forster et al., 2013). The type II Na+-coupled Pi cotransporter (*SLC34A2*), also found in the brush border of the small intestine, is responsible for the majority of sodium-dependent P uptake, especially under low dietary P conditions (Sabbagh et al., 2011). Its expression and activity might also be upregulated in response to P deficiency (Ren et al., 2020). Both *SLC20A1* and *SLC20A2* are involved in the active P transport across the intestinal epithelium, with *SLC20A1* being the main contributor among the two (Keasey et al., 2016). Their function is to transport P from the intestinal cavity into the enterocyte (Lv et al., 2022). Zechner et al., (2022) reported that *SLC20A1* showed lower protein expression in phosphate-repleted human HEK293T cells but significantly increased following phosphate starvation, suggesting its essential role in cellular P uptake under conditions of low P supply. In line with our results, Huber et al., (2015) reported a decreased ileal expression of *SLC20A1* in broilers fed a high P diet. Several studies also confirmed the activation of transcellular P transporters in the small intestine of both laying hens and broiler chickens fed low P diets, suggesting that intestinal cells respond to the amount of supplied P through endocrine signals such as calcitriol (Rousseau et al., 2016; Proszkowiec-Weglarz et al., 2019; Sommerfeld et al., 2020a; Omotoso et al., 2023). Passive mineral transport, including P, utilizes the paracellular pathway in mammals. This pathway involves proteins like claudin (*CLDN*), occludin (*OCLN*), and tight junction proteins (*TJP1, TJP2*, and *TJP3*) (Gloux et al., 2019). Tight junctions create a barrier between intestinal cells, and their permeability can influence P absorption indirectly. While the exact role of *TJP2* and *TJP3* in P transport needs further investigation, studies suggest that their expression might be modulated by dietary P levels (Gloux et al., 2019). Further research is needed to fully understand the role of *TJP2* and *TJP3* in P transport, particularly regarding their potential modulation by dietary P and its impact on paracellular permeability and P absorption.

In the context of comparing the expression profiles between the diets of each laying hen strain at the same time point, the metabolic pathways involved in the different gut functionality of the laying hens are of particular importance. At week 19, significant portions of the DEGs in the P diet groups in LB hens were notably enriched in metabolic pathways. However, by 24 weeks, these changes diminished, with the effect being less pronounced in the LSL strain. Since P is an essential element and plays a crucial role in various biological processes, especially during the growth phase, the reduced P content plays a more prominent role in metabolic processes at week 19 than week 24, especially in the LB strain. Higher transcript levels in P- groups in metabolic pathways and carbon metabolism suggest divergent physiological responses at the molecular level due to the complete removal of mineral P from the diet. The TCA cycle, also known as the citric acid cycle, is a central pathway for generating energy (ATP) from carbohydrates, fats, and proteins. A recent study reported that the gut is a major contributor to circulating TCA cycle metabolites (Tong et al., 2022). The effects of a P- diet on the TCA cycle and other metabolic pathways may trigger metabolic adaptations aimed at optimizing nutrient utilization and conserving P. Interestingly, in LB strains, we observed lower expression of genes associated with the immune system (cytokine-cytokine receptor interaction and influenza A), suggesting a possible trade-off between metabolic adaptation and immune function in response to lack of mineral P. As also reported by other studies, hens might need to adjust their metabolism to maintain health and production performance in response to a P deficient diet (Nie et al., 2018). The LB strain exhibited a notable response at week 19 to the withdrawal of dietary mineral P supplementation, showing increased expression of genes associated with metabolic pathways and decreased expression of genes associated with immune pathways. Moreover, reducing the P in the diet for four weeks (20-24 weeks) resulted in fewer molecular changes in the gut compared to the four-week maturation phase (15-19 weeks), despite no change in hen body weight based on the diet at both time points (Sommerfeld et al., 2024).

To conclude, among the factors studied, strain had the strongest impact, with genetic differences between the LB and LSL strains shaping gene expression patterns. The maturation period played a secondary role within each strain, with a shift from immune function genes at 19 weeks, preparing for egg production, to genes related to egg formation and energy metabolism after the onset of laying at 24 weeks. Dietary P content had the least pronounced effect, with changes in genes related to P transport and utilization being less conspicuous compared to strain and age effects. Notably, the LB strain at 19 weeks exhibited a heightened response to the removal of dietary mineral P supplementation, showing increased expression of metabolic transcripts and reduced immune transcripts. Reduced molecular changes were observed at 24 weeks, particularly in LSL hens. While this study found no adverse effects of removing mineral P supplementation in four-week periods in matured laying hens, the observed strain-specific differences in molecular patterns challenge the current one-size-fits-all approach to recommended P levels in the diet of laying hens.

## Ethics declarations

The experiment was conducted at the agricultural experiment station of the University of Hohenheim, Germany, with the approval of the regional council of Tübingen, approval number HOH67/21TE, in accordance with the German animal welfare legislation.

## Disclosures

All authors declare no conflicts of interests.

## Data Availability

Expression data are deposited in the ArrayExpress (Accession number: E-MTAB-14047 and will be released on: 2024-12-31). Reviewer's share link: https://www.ebi.ac.uk/biostudies/arrayexpress/studies/E-MTAB-14040?key=e690ec54-d86b-4fcf-bc15-62e60fdc6eaf

## Appendix Supplementary materials

Image, application 1
